# Predictors of the chest CT score in COVID-19 patients: a cross-sectional study

**DOI:** 10.1186/s12985-021-01699-6

**Published:** 2021-11-18

**Authors:** Niloofar Ayoobi Yazdi, Abdolkarim Haji Ghadery, Seyed Ahmad Seyedalinaghi, Fatemeh Jafari, Sirous Jafari, Malihe Hasannezad, Hamid Emadi Koochak, Mohammadreza Salehi, Seyed Ali Dehghan Manshadi, Mohsen Meidani, Mahboubeh Hajiabdolbaghi, Zahra Ahmadinejad, Hossein Khalili, Mohammad-Mehdi Mehrabinejad, Ladan Abbasian

**Affiliations:** 1 Department of Radiology, Imam Khomeini Hospital, Tehran University of Medical Sciences, Tehran, Iran; 2 Department of Radiology, Advanced Diagnostic and Interventional Radiology Research Center(ADIR), Tehran University of Medical Sciences, Tehran, Iran; 3 Iranian Research Center for HIV/AIDS, Iranian Institute for Reduction of High-Risk Behaviors, Tehran University of Medical Sciences, Tehran, Iran; 4 Department of Infectious Diseases, Imam Khomeini Hospital, Imam Khomeini Hospital Complex, Tehran University of Medical Sciences, Blv. Keshavarz, Tehran, Iran; 5 Department of Pharmacotherapy, Imam Khomeini Hospital Complex, Tehran University of Medical Sciences, Tehran, Iran

**Keywords:** COVID-19, Computed tomography, Outcome, Recovery, Chest CT score

## Abstract

**Background:**

Since the COVID-19 outbreak, pulmonary involvement was one of the most significant concerns in assessing patients. In the current study, we evaluated patient’s signs, symptoms, and laboratory data on the first visit to predict the severity of pulmonary involvement and their outcome regarding their initial findings.

**Methods:**

All referred patients to the COVID-19 clinic of a tertiary referral university hospital were evaluated from April to August 2020. Four hundred seventy-eight COVID-19 patients with positive real-time reverse-transcriptase-polymerase chain reaction (RT-PCR) or highly suggestive symptoms with computed tomography (CT) imaging results with typical findings of COVID-19 were enrolled in the study. The clinical features, initial laboratory, CT findings, and short-term outcomes (ICU admission, mortality, length of hospitalization, and recovery time) were recorded. In addition, the severity of pulmonary involvement was assessed using a semi-quantitative scoring system (0–25).

**Results:**

Among 478 participants in this study, 353 (73.6%) were admitted to the hospital, and 42 (8.7%) patients were admitted to the ICU. Myalgia (60.4%), fever (59.4%), and dyspnea (57.9%) were the most common symptoms of participants at the first visit. A review of chest CT scans showed that Ground Glass Opacity (GGO) (58.5%) and consolidation (20.7%) were the most patterns of lung lesions. Among initial clinical and laboratory findings, anosmia (*P* = 0.01), respiratory rate (RR) with a cut point of 25 (*P* = 0.001), C-reactive protein (CRP) with a cut point of 90 (*P* = 0.002), white Blood Cell (WBC) with a cut point of 10,000 (*P* = 0.009), and SpO_2_ with a cut point of 93 (*P* = 0.04) was associated with higher chest CT score. Lung involvement and consolidation lesions on chest CT scans were also associated with a more extended hospitalization and recovery period.

**Conclusions:**

Initial assessment of COVID-19 patients, including symptoms, vital signs, and routine laboratory tests, can predict the severity of lung involvement and unfavorable outcomes.

## Background

The coronavirus disease (COVID-19), caused by a novel coronavirus named severe acute respiratory syndrome coronavirus2 (SARS-CoV-2), has spread to 223 countries with more than 186 million confirmed cases and more than 4 million deaths (World Health Organization) [1]. SARS-CoV-2 shares similarities in disease dynamics, transmission route, and cell entry receptors, angiotensin-converting enzyme 2 (ACE2) with severe acute respiratory syndrome coronavirus (SARS-CoV) [2–4].

Person-to-person transmission of SARS-CoV-2 occurs with respiratory droplets from an infected person to others. Viral shedding may occur 1–2 days before the onset of symptoms and may continue for 1–2 weeks in mild to moderate cases or go beyond two weeks in severe cases[5, 6]. Symptoms usually appear between 2 and 14 days after exposure; The common initial symptoms of COVID-19 are fever, cough, fatigue, and dyspnea [7, 8], with more specific symptoms, including loss of taste and smell [6]. SARS-CoV-2 targets multiple organs, including respiratory, cardiac, and renal systems causing pneumonia and respiratory failure in patients [9, 10]. In addition, systemic inflammatory response syndrome and cytokine storm contribute to multi-organ failure and coagulopathy in critical patients with COVID-19 [11, 12].

Computed tomography (CT) is the most sensitive tool for diagnosing COVID-19, and several radiological patterns are seen in different phases of disease [13, 14]. Among different patterns of chest CT scan, ground-glass opacities (GGO) and mixed GGO with consolidation is reported as the most common patterns in COVID-19 patients [15]. Although definite diagnosis relies on real-time reverse-transcriptase-polymerase chain reaction (RT-PCR) [16], chest CT is a valuable modality to measure the extent of lung involvement and propose a treatment plan.

Initial assessment of COVID-19 patients is essential for further management. In this study, we assessed the patient’s signs, symptoms, laboratory tests, and imaging findings to identify which initial clinical and laboratory findings may predict the severity of lung involvement and, accordingly, short-term outcome.

## Method

### Study design and participants

The study protocol and consent notes were reviewed and approved by the ethics board of our institute (approval code: TUMS.VCR.REC.1399.138). This study is a cross-sectional, observational, single-center study conducted between April and August 2020 at a tertiary medical center. All referred patients to the COVID-19 clinic of Imam Khomeini Hospital, university hospital, were evaluated for eligibility of participation in the study. Patients with mild, moderate, or severe clinical symptoms suggestive of COVID-19 who had positive RT-PCR (226, 47.2%) confirming COVID-19 or suggestive chest CT scan (252, 52.8%) were included after signing a written informed consent form. Patients with history of previous lung diseases excluded from study. Four physicians did the patient examination and data registration, and five medical residents followed up with the patients through telephone calls. Both hospitalized (after discharge) and outpatient participants were followed weekly through telephone calls until symptoms resolved. Treatment regimens were based on the latest version of the national protocol of COVID-19.Hydroxychloroquine was used as the primary therapeutic option in the outpatient setting; hydroxychloroquine, lopinavir/ritonavir (Kaletra), atazanavir (nonboosted with ritonavir or cobicistat) were administered for hospitalized patients, and in case of severe hypoxemia (not attaining SpO2 of > 88% with reservoir mask), corticosteroid was given.

### Data collection

Following clinical data were collected in the specific forms for each patient*,* including: (a) *demographics information*: (age and sex); (b) *vital signs*: temperature (T-Celsius), oxygen saturation (SpO_2_), respiratory rate (RR per minute), and pulse rate (PR per minute); (c) *symptoms*: myalgia, generalized weakness, fever, chills, headache, chest pain, dyspnea, sore throat, cough, sputum, loss of appetite, loss of taste, anosmia; (d) *comorbidities*: hypertension (HTN), diabetes mellitus (DM); (e) *laboratory data*: white blood cell (WBC-cell/mm3), lymphocyte count (cell/mm3), platelet(cell/mm3), C-reactive protein (CRP-mg/L), erythrocyte sedimentation rate (ESR-mm/hr), and lactate dehydrogenase (units/L); (f) *admission status:* inpatient or outpatient; (g) *Intensive care unit (ICU) admission*; (h) *death*; (i) *radiologic findings* (will be mentioned in following section); (j) *length of hospitalization (day) and recovery time (day).* The recovery time was defined as the patient's subjective statement indicating no symptoms other than his/her baseline.

### Chest CT protocols and interpretation

All chest CT scans were performed on either lightspeed 64-detector CT (GE Healthcare) or the 16-slice (Siemens SOMATOM Emotion) MDCT scanner with patients in the supine position at full inspiration breath-hold. The leading scanning parameters were as follows: 120 kVp tube voltage; 50–150 mAs tube current; 0.75 s tube rotation time; 0.5–0.75 s gantry rotation time; 2–3-mm section thickness; and 0.6–2 mm beam collimation.

The visual chest CT interpretation was performed using a single, experienced (10 years) thoracic radiologist. The radiologist was blinded to clinical and laboratory data of participants and reviewed on both lung and mediastinal window settings. The presence of CT features including (a) *predominant pattern of lesions:* ground-glass opacification (GGO), consolidation, or mixed GGO and consolidation; (b) *dominant distribution of lesions:* central, peripheral, diffuse, or peri- broncho vascular; (c) *shape of lesions*: round, elongated, wedged, or confluent; (d) *additional findings:* crazy paving pattern*,* reverse-halo sign, interlobular septal thickening, linear opacities combined, air bronchogram sign, tree in bud*,* adjacent pleural thickening, pleural effusion, pericardial effusion, lymphadenopathy, and pulmonary emphysema, was reported as defined in Fleischner Society Glossary of terms for Thoracic Imaging [17].

To quantify the extension of lung lesions, a scoring system as follows was used: each of the five lobes of lungs visually scored from 0 to 5 (0, no involvement; 1, < 5% involvement; 2, 5–25% involvement; 3, 26–49% involvement; 4, 50–75% involvement; 5, > 75% involvement). Then, the total chest CT score was calculated by the sum of each lob's scores, ranging from 0 to 25 [18].

### Statistical analysis

The statistical analysis was performed using SPSS version 16 (SPSS Inc. Chicago, IL). Continuous variables were presented as mean (standard deviation), and categorical variables as frequency and percentages. Variables were tested for normality using Kolmogorov–Smirnov test. Normally distributed continuous variables were analyzed using the independent sample *t*-test; otherwise, the Mann–Whitney *U* test was used. A Chi-square test was employed for nominal variables. *P* values less than 0.05 were considered statistically significant.

## Results

### Participant’s characteristic

A total of 478 participants were recruited in the current study with convenient sampling. Of those, 353 (73.6%) participants were admitted to the hospital, and among hospitalized participants, 42 (8.7%) were admitted to ICU. The patient's mean age was 53.92 (15.4), and 267 (55.8%) was male. The most common complaints of patients were myalgia (60.4%), fever (59.4%), dyspnea (57.9%), chills (49.5%), and generalized weakness (45.3%). Demographics, initial signs, symptoms, and laboratory data of all participants are listed (Table 1).

**Table 1:** Detail of demographic data and baseline signs, symptoms, and laboratory findings of all studied participants

Variables	All patients N = 478
*Demographics*	
Age (years)	53.92(15.4)
< 39	90 (18.8%)
39–49	102 (21.3%)
50–59	104 (21.7%)
60–69	98 (20.5%)
≥ 70	84 (17.5%)
Gender (male)	267 (55.8%)
*Symptoms on the first visit*	
Myalgia	289 (60.4%)
Generalized weakness	217 (45.3%)
Fever	284 (59.4%)
Chills	237 (49.5%)
Headache	134 (28%)
Chest pain	126 (26.3%)
Dyspnea	277 (57.9%)
Sore throat	80 (16.7%)
Cough	334 (69.8%)
Sputum	15 (3.1%)
Loss of appetite	194 (40.5%)
Loss of taste	48 (10%)
Anosmia	60 (12.5%)
*Vital signs on first visit*	
Temperature (°C)	
≤ 37.2	212 (44.3%)
≥ 37.3	266 (55.7%)
O2 saturation %	
≥ 93	276 (57.7%)
< 93	202 (42.3%)
Respiratory rate (breaths/minute)	
< 25	389 (81.3%)
≥ 25	89 (18.7%)
*Initial laboratory findings*	
White blood cell (WBC) (cell/ mm^3^)	
< 4000	51 (10.6%)
4000–9999	364 (76.3%)
≥ 10,000	63 (13.1%)
Lymphocyte count (cell/ mm^3^)	
≤ 1000	103 (21.5%)
> 1000	375 (78.5%)
Platelet (cell/mm^3^)	
< 150,000	82 (17.1%)
≥ 150,000	396 (82.9%)
C-reactive protein (CRP) (mg/L)	
≤ 90	17 (3.5%)
≥ 91	461 (96.5%)
Erythrocyte sedimentation rate (ESR) (mm/h)	
≤ 60	13 (2.7%)
≥ 61	465 (97.3%)
Lactate dehydrogenase (LDH) (units/L)	
< 480	65 (13.5%)
≥ 480	413 (86.5%)
Underlying disease	
DM	132 (27.6%)
HTN	136 (28.4%)

All variables are reported as N (%)

HTN: hypertension, DM: diabetes mellitus

### Chest CT scan findings

The most common patterns of pulmonary involvement were GGO (58.5%) and consolidation 99 (20.7%) with peri-broncho-vascular (33.7%), peripheral (33%), and diffuse (32%) distribution, and the most common shape of lesions were confluent (47.2%). Detailed chest CT scan findings are presented (Table 2).

**Table 2:** Radiological findings in all studied participants

Variables	All patients N = 478
Pulmonary involvement scores*	
RUL Total Score	1.57 (1.75)
RML Total Score	1.26 (1.71)
RLL Total Score	1.91 (1.84)
LUL Total Score	1.56 (1.72)
LLL Total Score	1.79 (1.84)
Total pulmonary involvement Score	8.11 (8.08)
Frequency of lobe involvement	
RUL	266 (55.5%)
RML	225 (47%)
RLL	304 (63.5%)
LUL	274 (57.3%)
LLL	287 (60%)
Laterality of lung involvement	
Unilateral	52 (10.8%)
Bilateral	286 (59.8)
Pattern of lesions	
GGO	280 (58.5%)
Consolidation	99 (20.7%)
Mixed GGO and consolidation	78(16.3%)
Dominant distribution of lesions	
Peripheral	158 (33%)
Central	6 (1.3%)
Diffuse	153 (32%)
Peri broncho vascular	161 (33.7%)
Shape of lesions	
Round	73 (15.2%)
Elongated	82 (17.1%)
Wedged	183 (38.2%)
Confluent	226 (47.2%)
Additional findings	
Crazy paving pattern	87 (31%)
Reverse-halo	14 (2.9%)
Interlobular septal thickening	20 (4.1%)
Linear opacities combined	81 (16.9%)
Air bronchogram sign	114 (23.8%)
Tree in bud	15 (3.1%)
Adjacent pleura thickening	15 (3.1%)
Pleural effusion	27 (5.6%)
Unilateral	12 (2.5%)
Bilateral	15 (3.1%)
Pericardial effusion	10 (2%)
Lymphadenopathy	7 (1.4%)
Pulmonary emphysema	10 (2%)

RUL, right upper lobe; RML, right middle lobe; RLL, right lower lobe; LUL, left upper lobe; LLL, left lower lobe

*Pulmonary involvement scores are reported as mean (standard deviation); all other variables are reported as N (%)

Our results showed that the total chest CT score was significantly higher in patients with anosmia (mean of 12.46 ± 7.73 vs. 8.73 ± 8.33; *P* = 0.01), and had a significant positive association with RR with a cut point of 25 (mean of 8.37 ± 7.94 vs. 12.37 ± 9.23; *P* = 0.001), CRP with a cut point of 90 (mean of 7.82 ± 7.89 vs. 10.83 ± 8.67; *P* = 0.002), WBC with a cut point of 10,000 (mean of 8.70 ± 8.33 vs. 12.02 ± 8.17; *P* = 0.009) and negative association with SpO2 with a cut point of 93 (mean of 9.75 ± 8.54 vs. 7.76 ± 7.58; *P* = 0.04). Chest CT score was also associated with a higher risk of ICU admission (mean of 11.10 ± 9 vs. 7.71 ± 7.88; *P* = 0.003), longer hospital stay (mean of 9.08 ± 8.23 vs. 12 ± 9.21; *P* = 0.037) and recovery period (mean of 7.28 ± 8.02 vs. 9.29 ± 8.09; *P* = 0.009) (Table 3).

**Table 3:** Demographic, clinical, laboratory findings, and outcomes of patients with COVID-19 based on the total chest CT scan score

Variables	All patients = 478	Total CT score Mean ± SD	*P* value
*Demographic data*			
Age (years)			
< 39	90 (18.8%)	10.47 ± 8.02	0.54^a^
39–49	102 (21.3%)	8.15 ± 8.19	
50–59	104 (21.7%)	9.96 ± 9.03	
60–69	98 (20.5%)	9.57 ± 8.23	
≥ 70	84 (17.5%)	8.44 ± 8.04	
Sex			
Male	267 (55.8%)	7.70 ± 7.84	0.193
Female	211 (44.2%)	8.67 ± 8.37	
*Symptoms on first visit*			
Fever			
Yes	284 (59.4%)	9.06 ± 8.25	0.78
No	194 (40.6%)	9.34 ± 8.53	
Dyspnea			
Yes	277 (57.9%)	9.56 ± 8.58	0.20
No	201 (42.1%)	8.38 ± 7.82	
Sputum			
Yes	15 (3.1%)	9.64 ± 9.70	0.87
No	463 (96.9%)	9.14 ± 8.30	
Cough			
Yes	334 (69.8%)	9.10 ± 8.20	0.85
No	144 (30.2%)	9.29 ± 8.68	
Chest pain			
Yes	126 (26.3%)	9.42 ± 8.08	0.74
No	352 (73.7)	9.07 ± 8.43	
Anosmia			
Yes	60 (12.5%)	12.46 ± 7.73	0.01*
No	418 (87.5%)	8.73 ± 8.33	
*Vital signs on first visit*			
Temperature (°C)			
≤ 37.2	212 (44.3%)	9.00 ± 8.37	0.76
≥ 37.3	266 (55.7%)	9.28 ± 8.35	
Respiratory rate(breath/minute)			
< 25	389 (81.3%)	8.37 ± 7.94	0.001*
≥ 25	89 (18.7%)	12.37 ± 9.23	
SpO2%			
≥ 93	276 (57.7%)	7.76 ± 7.58	0.04*
< 93	202 (42.3%)	9.75 ± 8.54	
*Initial laboratory findings*			
WBC (cell/ mm^3^)			
≤ 10,000	415 (86.9%)	8.70 ± 8.33	0.009*
> 10,000	63 (13.1%)	12.02 ± 8.17	
Lymphocyte count(cell/ mm^3^)			
≤ 1000	103 (21.5%)	9.77 ± 8.88	0.42
> 1000	375 (78.5%)	8.91 ± 8.07	
CRP (mg/L)			
≤ 90	17 (3.5%)	7.82 ± 7.89	0.002*
91	461 (96.5%)	10.83 ± 8.67	
ESR(mm/h)			
≤ 60	13 (2.7%)	8.24 ± 8.45	0.10
≥ 61	465 (97.3%)	10.05 ± 8.54	
LDH (units/L)			
< 480	65 (13.5%)	9.02 ± 7.92	0.12
≥ 480	413 (86.5%)	11.02 ± 8.64	
*Underlying disease*			
DM			
Yes	132 (27.6%)	8.54 ± 8.10	0.35
No	346 (72.4%)	9.42 ± 8.45	
HTN			
Yes	136 (28.4%)	9.11 ± 8.30	0.92
No	342 (71.6%)	9.22 ± 8.36	
*Outcomes*			
ICU admission			
Yes	42 (8.7%)	11.10 ± 9	0.003*
No	436 (92.2%)	7.71 ± 7.88	
Recovery time (days)			
< 15	278 (58.1%)	7.28 ± 8.02	0.009*
≥ 15	200 (41.9%)	9.29 ± 8.09	
Hospitalization time (days)			
< 15	437 (91.4%)	9.08 ± 8.23	0.037*
≥ 15	41 (8.6%)	12 ± 9.21	
Death			
Yes	16 (3.3%)	8.51 ± 1.85	0.86
No	462 (96.7%)	8.05 ± 0.37	

HTN, hypertension; DM, diabetes mellitus; SpO2, oxygen saturation; WBC, white blood cell; LDH, lactate dehydrogenase; CRP, C-reactive protein; ESR, erythrocyte sedimentation rate; ICU, intensive care unit

*Statistically significant (*p* value < 0.05)

^a^One way ANOVA test is used; independent t-test or Mann–Whitney U test used for all other comparisons

Further analysis showed consolidation on chest CT scan had a significant association with initial lower SpO2 (*P* = 0.001), higher risk of ICU admission (*P* = 0.005), extended hospitalization (*P* = 0.003), and longer recovery time (*P* = 0.008) (Table 4).

**Table 4:** Details of demographic and clinical data of patients with or without consolidation

Variables	All patients N = 478	With consolidation N = 99	Without consolidation N = 379	*P* value
*Demographic data*				
Age	53.92 (15.4)	51.96 (15.6)	54.62 (15.6)	0.14
Gender				
Male	267 (55.8%)	55 (11.5%)	212 (44.3%)	0.94^a^
Female	211 (44.2%)	44 (20.8%)	167 (23.4%)	
*Clinical data*				
Temperature (°C)	37.49 (0.7)	37.56 (0.7)	37.47 (0.7)	0.29
Respiratory rate (breath /per minute)	21.65 (5.1)	22.18 (6)	21.41 (4.9)	0.19
SpO2	91.07 (5.4)	89.39 (6.4)	91.47 (5.3)	0.001*
WBC (cell/mm^3^)	7.74 (5.1)	7.88 (5.6)	7.29 (4.2)	0.32
Lymphocyte count(cell/mm^3^)	1372.8 (1341.3)	1375.5 (1876.5)	1279 (870)	0.56
LDH (U/L)	640.8 (302.4)	624.6 (266.4)	630.1 (302.4)	0.90
CRP (mg/L)	96.90 (76.2)	105.97 (69.6)	90.82 (77)	0.13
ESR (mm/h)	74.06 (32.2)	77.34 (33.3)	71.23 (31.2)	0.16
Underlying disease				
DM	132 (27.6%)	26 (5.4%)	106 (22.2%)	0.73^a^
HTN	136 (28.4%)	23 (4.8%)	113 (23.6%)	0.18^a^
Recovery time (days)	15.48 (8.3)	17.46 (9)	14.95 (8.1)	0.008*
Hospitalization duration (days)	6.89 (7.2)	8.97 (9.4)	6.25 (6.2)	0.003*
ICU admission	42 (8.7%)	20 (20.2%)	22 (5.5%)	0.005*^a^
Death	16 (3.3%)	4 (0.8%)	12 (2.5%)	0.70^a^

HTN, hypertension; DM, diabetes mellitus; SpO2, oxygen saturation; WBC, white blood cell; LDH, lactate dehydrogenase; CRP, C-reactive protein; ESR, erythrocyte sedimentation rate; ICU, intensive care unit

*Statistically significant (*p* value < 0.05), reported as mean (standard deviation), all other variables reported as N (%)

^a^Chi-squared test is used; independent t-test or Mann–Whitney U test used for all other comparisons

## Discussion

As treatment protocols are based on the extent of lung involvement, assessing the severity of pulmonary involvement is crucial in determining the treatment plan for COVID-19 infected patients. Chest CT scan is not available everywhere, and its usage is sometimes limited, especially in pregnant patients. Therefore, physicians need prompt assessment according to patient’s initial signs and symptoms and routine laboratory tests for timely management. In the current study, we assessed clinical and laboratory findings on the first visit to predict the extent of lung involvement in COVID-19 patients. We found that patients with anosmia, lower SpO2 (< 93), and higher respiratory rate (> 25 per minute), WBC count (> 10,000 cells/mm3), CRP (> 90 mg/Liter) had higher total CT scores. Besides, consolidation opacities as the worst lung lesions were more commonly detected in patients with lower initial SpO2 or admitted to ICU. Moreover, patients with consolidation on their chest CT scan experienced extended hospitalization (≥ 15) and recovery period (≥ 15). A higher chest CT score was also associated with more extended hospitalization and recovery time.

### Predictors of severe lung involvement

In line with previous studies, our results showed that higher initial WBC and CRP are associated with more severe cases of COVID-19 patients, as WBC > 1000 cell/mm3 and CRP > 90 mg/L were associated with higher chest CT scores. Similar to our findings, Salvatore et al. reported hospitalized and critical COVID-19 patients had higher CRP, leukocyte count, neutrophils, LDH, D-dimer, and troponin [19]. Zhang et al. also reported that chest CT score positively associated with WBC count, CRP, ESR, procalcitonin, and abnormal coagulation function [20].

Among several clinical symptoms on the first visit, just anosmia was associated with extended pulmonary involvement. A literature review did not show any previous report of extended lung involvement in patients with anosmia, and our study is the first to report this correlation. Association of anosmia and pulmonary involvement can be justified by the roll ofchemokines, as previous reports defined CXCL10 contribution in both cytokine storm of COVID-19 patients causing acute respiratory distress syndrome (ARDS) and demyelination process of the olfactory nerve causing anosmia [21].

Several clinical signs of patients on the first visit, including higher RR and lower SpO2, were associated with higher total chest CT scores. Lower SpO2 was also associated with consolidation opacities as the most severe lesion of COVID-19 in chest CT scan. In line with our findings, Kunwei Li et al. also showed that patients with RR ≥ 30 times/min, and SpO2 ≤ 93% as categorized in severe type patients, had significantly higher total chest CT scores than common type patients [22]. Another study also confirmed that severe/critical patients with respiratory rate ≥ 30 times/min and SpO2 of 93% or less in a resting state had higher total CT scores than ordinary COVID-19 patients [23]. Aalinezhad [24] and Osman[25] et al. also reported higher chest CT score is inversely associated with O2 saturation. Furthermore, a multicenter cohort study demonstrated that consolidation in upper lungs on the initial chest CT of COVID-19 patients was associated with increased odds of adverse endpoints, including SpO2 < 93% and partial arterial pressure of oxygen less than 60 mm Hg on room air [26].

### Chest CT predictors of unfavorable outcomes

We found that ICU admission, more extended hospitalization, and recovery period were associated with higher total CT scores and consolidation opacities in chest CT of these patients. Similarly, a previous study showed a higher pulmonary score ≥ of 8 accompanied by age ≥ 53, and SpO_2_ ≤ 91 predicts ICU admission and mortality [27]. Deepak Nagra et al. also reported a higher lung opacification score is a reliable predictor of ICU admission for COVID-19 patients [28]. Another study on baseline chest CT scans and clinical and laboratory data of 72 patients admitted with COVID-19 pneumonia showed lung severity score >  was associated with a significantly lower recovery rate and discharge and extended hospitalization in patients admitted for COVID-19 pneumonia [29]. Similar to our findings, Ahlstrand et al. previously reported that chest CT score at hospital admission correlates closely with hospital length of stay and ICU admission [30]. CT severity score combined with age and history of at least one underlying disease had 79.7% sensitivity and 65.5% specificity in predicting the adverse outcomes in a previous study [31].

Initial assessment of patients in the clinic determines the treatment plan, so we assessed lung involvement based on initial signs, symptoms, and laboratory tests. Taken together, we found that patients with anosmia, higher respiratory rate, WBC count, or CRP, and lower SpO2 had extended pulmonary involvement of COVID-19 pneumonia, which can cause adverse outcomes, including more extended hospitalization and recovery period. Hence, those patients should be prioritized for greater attention and intensive care.

### Limitations

As recovery time was defined as a subjective statement of patients, this could have resulted in a conclusion error; inviting the patients to the clinic for evaluation could have been more precise to confirm recovery. In addition, in this study, we assessed just the presence of symptoms, not their severity; we recommend further studies assessing the severity of symptoms and their correlation with lung involvement or adverse outcomes.

## Conclusion

Extended lung involvement of COVID-19 pneumonia can be predicted in clinics with patient's initial symptoms, vital signs, and laboratory tests, including anosmia, low SpO2, high RR, WBC, and CRP. Therefore, these patients should be considered high-risk patients for further medical planning.

## Data Availability

The datasets used and/or analysed during the current study are available from the corresponding author on reasonable request.

## Abbreviations

COVID-19: The coronavirus disease
SARS-CoV-2: Severe acute respiratory syndrome coronavirus2
ACE2: Angiotensin-converting enzyme 2
SARS-CoV: Severe acute respiratory syndrome coronavirus
CT: Computed tomography
GGO: Ground glass opacity
RT-PCR: Real-time reverse-transcriptase-polymerase chain reaction
SpO_2_: Oxygen saturation
RR: Respiratory rate
PR: Pulse rate
HTN: Hypertension
DM: Diabetes mellitus
WBC: White blood cell
CRP: C-reactive protein
ESR: Erythrocyte sedimentation rate
LDH: Lactate dehydrogenase
ICU: Intensive care unit
ARDS: Acute respiratory distress syndrome
RUL: Right upper lobe
RML: Right middle lobe
RLL: Right lower lobe
LUL: Left upper lobe
LLL: Left lower lobe
