# Correction to: Predictors of the chest CT score in COVID‑19 patients: a cross‑sectional study

**DOI:** 10.1186/s12985-021-01719-5

**Published:** 2021-12-06

**Authors:** Niloofar Ayoobi Yazdi, Abdolkarim Haji Ghadery, SeyedAhmad SeyedAlinaghi, Fatemeh Jafari, Sirous Jafari, Malihe Hasannezad, Hamid Emadi Koochak, Mohammadreza Salehi, Seyed Ali Dehghan Manshadi, Mohsen Meidani, Mahboubeh Hajiabdolbaghi, Zahra Ahmadinejad, Hossein Khalili, Mohammad-Mehdi Mehrabi Nejad, Ladan Abbasian

**Affiliations:** 1 Department of Radiology, Imam Khomeini Hospital, Tehran University of Medical Sciences, Tehran, Iran; 2 Department of Radiology, Advanced Diagnostic and Interventional Radiology Research Center(ADIR), Tehran University of Medical Sciences, Tehran, Iran; 3 Iranian Research Center for HIV/AIDS, Iranian Institute for Reduction of High-Risk Behaviors, Tehran University of Medical Sciences, Tehran, Iran; 4 Department of Infectious Diseases, Imam Khomeini Hospital, Imam Khomeini Hospital Complex, Tehran University of Medical Sciences, Blv. Keshavarz, Tehran, Iran; 5 Department of Pharmacotherapy, Imam Khomeini Hospital Complex, Tehran University of Medical Sciences, Tehran, Iran

## Correction to: Virology Journal (2021) 18:225 10.1186/s12985-021-01699-6

Following publication of the original article [1], the authors identified an error in the author name of SeyedAhmad SeyedAlinaghi and in the author name of Mohammad‑Mehdi Mehrabi Nejad.

The incorrect author name is: Seyed Ahmad Seyedalinaghi

The correct author name is: SeyedAhmad SeyedAlinaghi

The incorrect author name is: Mohammad-Mehdi Mehrabinejad

The correct author name is: Mohammad‑Mehdi Mehrabi Nejad

The original article has been corrected.
